# Spatiotemporal analysis of food insecurity in Iran from 2013 to 2023

**DOI:** 10.1038/s41598-026-52946-1

**Published:** 2026-05-13

**Authors:** Ali Goli, Mehrdad Askarian

**Affiliations:** 1 Department of Sociology and Social Planning, Faculty of Economics, Management and Social Sciences, Shiraz University, Shiraz, Iran; 2 Department of Community Medicine, Shiraz Medical School, Shiraz University of Medical Sciences, Shiraz, Iran

**Keywords:** Food Insecurity, Spatiotemporal analysis, FGT indices, Iran Counties, Geography, Geography, Health care

## Abstract

Food is a fundamental human necessity, and shortages or insufficient caloric intake led to food insecurity, with consequences that extend from the individual to the societal level. In Iran, various socioeconomic changes in recent years have contributed to a steady rise in food insecurity, resulting in a significant increase in its prevalence across most regions of the country. This study investigates the spatiotemporal patterns of food insecurity using Foster–Greer–Thorbecke (FGT) indices applied to annual Household Income and Expenditure Survey (HIES) data from 2013 to 2023. Moran’s I and Local Indicators of Spatial Association (LISA) are employed to analyze spatial clustering and spatiotemporal dynamics in conjunction with FGT measures of prevalence, gap, and severity. The dataset consists of annual observations from approximately 38,000 sample households in urban and rural areas of Iran, analyzed through spatial data mining at the county level. Findings indicate that in 2013, approximately 33% of surveyed households experienced food insecurity, largely concentrated in specific regions. In contrast, during the final three years of the study period—following the 2018 and 2021 removal of subsidies from energy and four major staple food groups in Iranian household consumption (flour, dairy products, poultry and eggs, and cooking oil)—the prevalence exceeded 49% and expanded across most regions of the country. If these conditions persist, particularly in the absence of policy support for essential food items within household consumption baskets, food insecurity is expected to intensify and become more widespread. Such a trajectory may pose substantial risks to public health, especially in already vulnerable regions. Furthermore, the food insecurity gap—based on a minimum daily caloric requirement of 2,100 calories—increased from 8.3% in 2013 to more than 14% in 2022 and 11.4% in 2023. Similarly, the severity of food insecurity, which reflects the extent of deprivation among the most affected households, rose by 35% over the decade. These results highlight critical spatial disparities that underscore the need for geographically targeted policy interventions.

## Introduction

Food security remains a critical global challenge, encompassing four essential dimensions: availability, access, utilization, and stability. In 2024, approximately 2.285 billion people (28% of the global population) faced moderate or severe food insecurity. Although Sustainable Development Goal 2 (SDG 2) aims for zero hunger by 2030, current projections indicate that the number of people affected by hunger could exceed 941 million by that year, with vulnerable populations facing heightened nutritional risks^1^.

According to cross-national estimates from the FAO, Iranian household food insecurity has declined somewhat, from 48% in 2014–2016 to 39% in 2022–2024, while severe food insecurity fell from 9.5% to 5.9% over the same period^2^. Nevertheless, over 35 million Iranians experienced severe food insecurity in 2024, highlighting the continued need for monitoring and targeted interventions within the framework of social welfare and public health. Notably, while macro-level supply estimates from the FAO suggest partial improvements, micro-level data reveal divergent trends that necessitate granular investigation^3^.

Household food insecurity has been extensively studied globally using measures such as the Foster–Greer–Thorbecke (FGT) index and the Household Food Insecurity Access Scale (HFIAS). For example, Malik and Shah^4^ reported over 31% prevalence of severe food insecurity in Gondar, Ethiopia; Arshad et al.^5^ observed 42% prevalence in Pakistan, with household size, limited education, and employment constraints as major determinants; and Omotayo et al.^6^ reported 58% prevalence among Nigerian farmers. Global prevalence rates range from 5.5% in Portugal^7^ to 22% in India’s Himalayan region^8^. Ren et al. evaluated the impacts of urbanization on land use changes in South China from 1990 to 2020, showing that urban area growth was primarily driven by the conversion of agricultural and forest lands into urban-built environments, and that protecting resources and promoting environmental sustainability is crucial for maintaining effective resources in agricultural production^9^. Bofa and Zewotir explored the spatiotemporal changes of factors affecting food security and nutrition in Africa using Principal Component Analysis (PCA) and longitudinal Generalized Poisson (LGP) models on FAO data from 2000 to 2019. They noted that despite the dynamic and evolving nature of factors influencing food security and nutrition—and numerous attempts to understand and reduce food insecurity—existing models often fail to capture this dynamism. Using a dynamic spatiotemporal Bayesian approach to explore the interconnected dynamics of food security and its components in Africa, they demonstrated a uniform pattern of high food security and nutrition levels that showed significant stability in early and mid-to-late stages, and then experienced remarkable acceleration in the final stage of the study period ^10-13^.

In Iran, studies demonstrate significant regional heterogeneity. Shabanzadeh Khoshrouyi et al. identified food security disparities in Tehran households^14^, while Ahmadi Dehrashid et al. reported that 80% of rural participants experienced food insecurity (25% mild, 42% moderate, 13% severe), with economic access as the primary determinant^15^. Rafati et al.^16^ documented high welfare inequality in Tehran. Bagheri et al.^17^ linked food insecurity to cognitive and relative poverty in rural Gilan. Other studies report prevalence rates between 24% and 74% across provinces and urban–rural contexts (Akbari et al.^18^; Akbarpour et al.^19^; Purtaheri et al.^20^; Najafi & Shushtarian^21^; Ramesh et al.^22^; Abdi et al.^23^; Ahmadi Javid et al.^24^; Azmayesh Asiabasri et al.^25^; Farshidi et al.^26^; Rostami et al.^27^; Shabanzadeh-Khoshrody et al.^28^; Shahraki & Ghader i^29^).

Despite this body of research, Iranian studies remain largely cross-sectional and micro-scale, with limited county-level or spatiotemporal analyses. A review of previous studies—particularly those examining food security in Iran—indicates that most analyses relied on periods shorter than five years and were conducted at a macro scale, often predating the supportive policy reforms implemented in 2022. Addressing these gaps requires comprehensive investigations that map food insecurity across both time and space, informing policies that enhance food access, reduce inequalities, and promote public health.

In response to these limitations, the present study adopts a more comprehensive and context-sensitive approach by analyzing a ten-year timeframe at the local (county) level and incorporating the post-reform period. Several critical drivers underscore the need for this approach: the removal of subsidies for several essential food items in 2022, the rising costs of major household expenditures such as housing and energy—particularly in large urban areas since 2019—and their subsequent effects on household food baskets. These factors represent critical drivers for identifying emerging dimensions of food insecurity across Iranian counties.

This study provides a unique contribution to the literature by offering the first decade-long, county-level spatiotemporal analysis of food insecurity in Iran. Unlike previous cross-sectional or macro-scale studies, this research specifically captures the impact of the 2022 subsidy reforms and rising costs of essential non-food expenditures—such as housing and energy—on household caloric intake at a high spatial resolution.

## Materials and methods

### Data

This study utilized data from the Statistical Center of Iran (SCI). HIES data, spanning 2013 to 2023, were obtained from urban and rural household surveys conducted annually by the SCI and were accessed for this study on January 15, 2026. (See Table 1 for sample distribution).

**Table 1:** Number of Sample Households and Individuals per Household (2013–2023).

Year	Sample household	Number of individuals in households
2013	38,421	140,359
2014	38,189	139,033
2015	38,143	137,616
2016	38,022	135,552
2017	37,860	134,389
2018	38,859	135,830
2019	38,196	132,451
2020	37,389	128,955
2021	37,826	128,914
2022	37,832	126,478
2023	37,883	123,857

The HIES data were collected using a standardized (COICOP^1^)-classified questionnaire. Consumer goods were categorized into 12 groups: (1) food and beverages, (2) tobacco, (3) clothing and footwear, (4) housing, water, electricity, gas, and other fuels, (5) furniture and household appliances, (6) health and medical care, (7) transport, (8) communications, (9) recreation and culture, (10) education, (11) hotels and restaurants, and (12) miscellaneous goods and services.

To assess food insecurity, household consumption of food and beverages was classified into nine groups:

1. **Cereals**: rice, wheat, corn, barley, bread, pasta, flour.
2. **Vegetables**: leafy, root, and starchy vegetables.
3. **Fruits**: citrus, berries, dried, and tropical fruits.
4. **Protein**: red meat, chicken, fish, and plant-based proteins (e.g., tofu, legumes).
5. **Dairy**: whole, low-fat, and non-fat products including milk, cheese, and yogurt.
6. **Fats and oils**: butter, margarine, vegetable oils, animal fats.
7. **Sugar and sweets**: sweets, chocolate, jam, syrups.
8. **Beverages**: alcoholic beverages, soft drinks, coffee, tea.
9. **Ready-made and processed foods**: snacks, pizza, sandwiches, soup^30^.

Calorie content was calculated using **U.S. Nutritional Data Center standards**, supplemented with tables detailing calories and protein content per food group. Per capita daily calorie intake was derived by dividing household calorie intake by household size. A minimum daily requirement of 2,100 kilocalories per individual was adopted as the reference standard. Households consuming below this threshold were identified, and the prevalence, gap, and severity of food insecurity were subsequently calculated^31^^,32^.

**Fig. 1:**
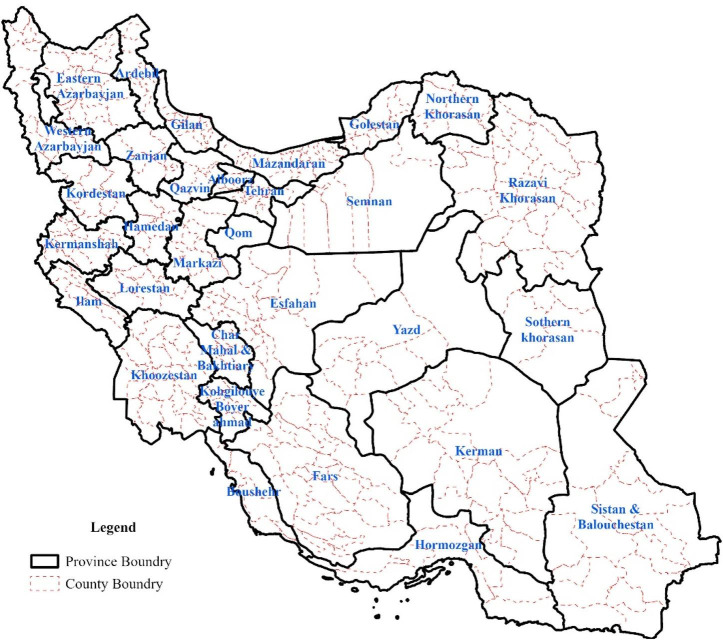
Iran provinces and counties in 2023: Iran Administrative boundary (31 Provinces & 431 Counties) in 2023^**30**^. Map digitized and created by using QGIS (version: 3.44) and Administrative Divisions Map of the Statistical Center of Iran, https://qgis.org/download/.

Iran’s current administrative divisions consist of 31 provinces and 484 counties. HIES data sampling was conducted according to the population distribution across urban and rural areas within these provinces and counties, ensuring representative coverage for spatial and household-level analyses (Fig. 1).

### Food insecurity measurement

The FGT index was used to assess food insecurity at household and individual levels. After calculating average daily caloric intake per individual, the index was applied to derive:

1 $$\:{\mathrm{F}\mathrm{I}}_{{\upalpha\:}}=\left(\frac{1}{\mathrm{N}}\right)\sum\:_{\mathrm{i}=1}^{\mathrm{q}}{\left[\frac{\mathrm{Z}-{\mathrm{Y}}_{\mathrm{i}}}{\mathrm{Z}}\right]}^{{\upalpha\:}}$$

The FGT indices— **Incidence** (**FI₀)**,** Food insecurity gap (FI₁)**, and **Severity** (**FI₂)** —offer a multidimensional picture of food insecurity:

1. **FI₀**: captured the proportion of households falling below the defined food security threshold. A high incidence indicates widespread vulnerability, suggesting that food insecurity is not confined to a limited set of households but is a structural issue. Proportion of households consuming less than 2,100 kcal/day.

2 $$\:{\mathrm{F}\mathrm{I}}_{0}=\left(\frac{\mathrm{q}}{\mathrm{N}}\right)$$

Where $\:{\mathrm{F}\mathrm{I}}_{0}$is the food insecurity index, N the population, q the number of food-insecure individuals.

2. **FI₁**: highlighted how far insecure households fall below the required expenditure or caloric threshold (α = 1). Regions with moderate FI₀ but high FI₁ point to deeply deprived households requiring intensive support.

3 $$\:{\mathrm{F}\mathrm{I}}_{1}=\left(\frac{1}{\mathrm{N}}\right)\sum\:_{\mathrm{i}=1}^{\mathrm{q}}{\left[\frac{\mathrm{Z}-{\mathrm{Y}}_{\mathrm{i}}}{\mathrm{Z}}\right]}^{\:}$$

That $\:{\mathrm{F}\mathrm{I}}_{1}$is the food insecurity gap, N the population, q the number of food-insecure individuals, Y_i_ individual caloric intake, Z the standard requirement.

3. **FI₂**: emphasized inequality among the food-insecure population, weighting the poorest more heavily. High severity values typically correspond to chronic under consumption, insufficient dietary diversity, and unstable income sources. These areas would benefit from long-term resilience programs rather than short-term food aid. Assigns higher weight to the most severely affected households (α = 2), following standard poverty measurement practices.

4 $$\:{\mathrm{F}\mathrm{I}}_{2}=\left(\frac{1}{\mathrm{N}}\right){\sum\:}_{\mathrm{i}=1}^{\mathrm{q}}{\left[\frac{\mathrm{Z}-{\mathrm{Y}}_{\mathrm{i}}}{\mathrm{Z}}\right]}^{2}$$

Equation 4 formalizes the index, where N the population, Y_i_ individual caloric intake, Z the standard requirement, q the number of food-insecure individuals, and α the severity weight (Eq. 4)^33^.

### Spatial analysis

FGT-derived values were linked to county-level GIS maps using Spatial Join to analyze spatiotemporal patterns:

- Spatial autocorrelation was computed using Moran’s I (global) and LISA (local) indices. We employed a first-order Queen Contiguity weight matrix, which was row-standardized to ensure that the sum of weights for each county equals one, allowing for robust comparison across administrative units.

5 $$\:\mathrm{I}=\:\frac{\mathrm{n}{\sum\:}_{\mathrm{i}=1}^{\mathrm{n}}{\sum\:}_{\mathrm{j}=1}^{\mathrm{n}}{\mathrm{w}}_{\mathrm{i}\mathrm{j}}\left({\mathrm{x}}_{\mathrm{i}}-\stackrel{-}{\mathrm{x}}\right)\left({\mathrm{x}}_{\mathrm{j}}-\stackrel{-}{\mathrm{x}}\right)}{\sum\:_{\mathrm{i}}\sum\:_{\mathrm{j}}{\mathrm{w}}_{\mathrm{i}\mathrm{j}}\sum\:_{\mathrm{i}}{\left({\mathrm{x}}_{\mathrm{i}}-\stackrel{-}{\mathrm{x}}\right)}^{2}}$$

Where:

N: Number of spatial units (Counties),

x_i_, xⱼ: Value of the variable in units *i* and *j*.

$\bar x$: Overall mean of the variable.

w_i_ⱼ: Spatial weight between units *i* and *j* (indicating adjacency or distance)^34^

- **LISA**: Identifies local spatial dependencies and heterogeneity, mapping hot spots (high-values) and cold spots (low-values)^35^.

6 $$\:{\mathrm{I}}_{\mathrm{i}}=\frac{\left({\mathrm{x}}_{\mathrm{i}}-\stackrel{-}{\mathrm{X}}\right)}{{m}_{2}}\sum\:_{\mathrm{j}}\left[{\mathrm{W}}_{\mathrm{i}\mathrm{j}}\left({\mathrm{x}}_{\mathrm{j}}-\stackrel{-}{\mathrm{X}}\right)\right]$$

Where $$\:\:{m}_{2}=\frac{1}{N}\sum\:_{k}{({x}_{k}-\stackrel{-}{x})}^{2}$$(7).

**Definitions**

- **xi**,** x**: Value of the variable at location *i*.
- $\:\stackrel{-}{\mathrm{x}}$: Mean value of the variable.
- **j**: All neighbors of unit *i*.
- **wi**: Spatial weight between units *i* and *j*.
- **N**: Number of spatial units.
- $\:{m}_{2}$: Variance term used for normalization.

Spatial patterns were interpreted according to Tobler’s First Law of Geography, which posits that nearby locations are more likely to influence each other. Differences may also reflect unique characteristics of individual locations^36^.

The combination of the FGT index and spatiotemporal analysis allowed for the identification of the intensity, prevalence, and depth of food insecurity, along with the spatiotemporal clustering and dispersion patterns, and enabled the detection of critical and exceptional areas. This analysis provides a basis for designing targeted, location-specific interventions and effective policy-making.

To examine the spatial patterns of food insecurity and their changes over the study period, Moran’s I was used to measure spatial autocorrelation at the county level. This index indicated how areas affected by food insecurity were arranged relative to each other—whether in clustered, dispersed, or random patterns.

Additionally, the LISA (Local Indicators of Spatial Association) index identified the local spatial structure of food insecurity, highlighting areas that simultaneously exhibited high intensity and spatial clustering.

FGT index tables for counties in 10 years joined as an attribute table to counties shapefile by County ID by QGIS software V3.44. The GeoDa software V1.22.0.20 (https://geodacenter.github.io/download) was used to detect Moran’s I (global) and LISA (local) indices as spatiotemporal changes over space and time among counties. Also, QGIS used to create thematic cartography. Both software are free and Open Source software that serves as free tools to spatial data science.

## Results

### Trends in food insecurity

**Fig. 2:**
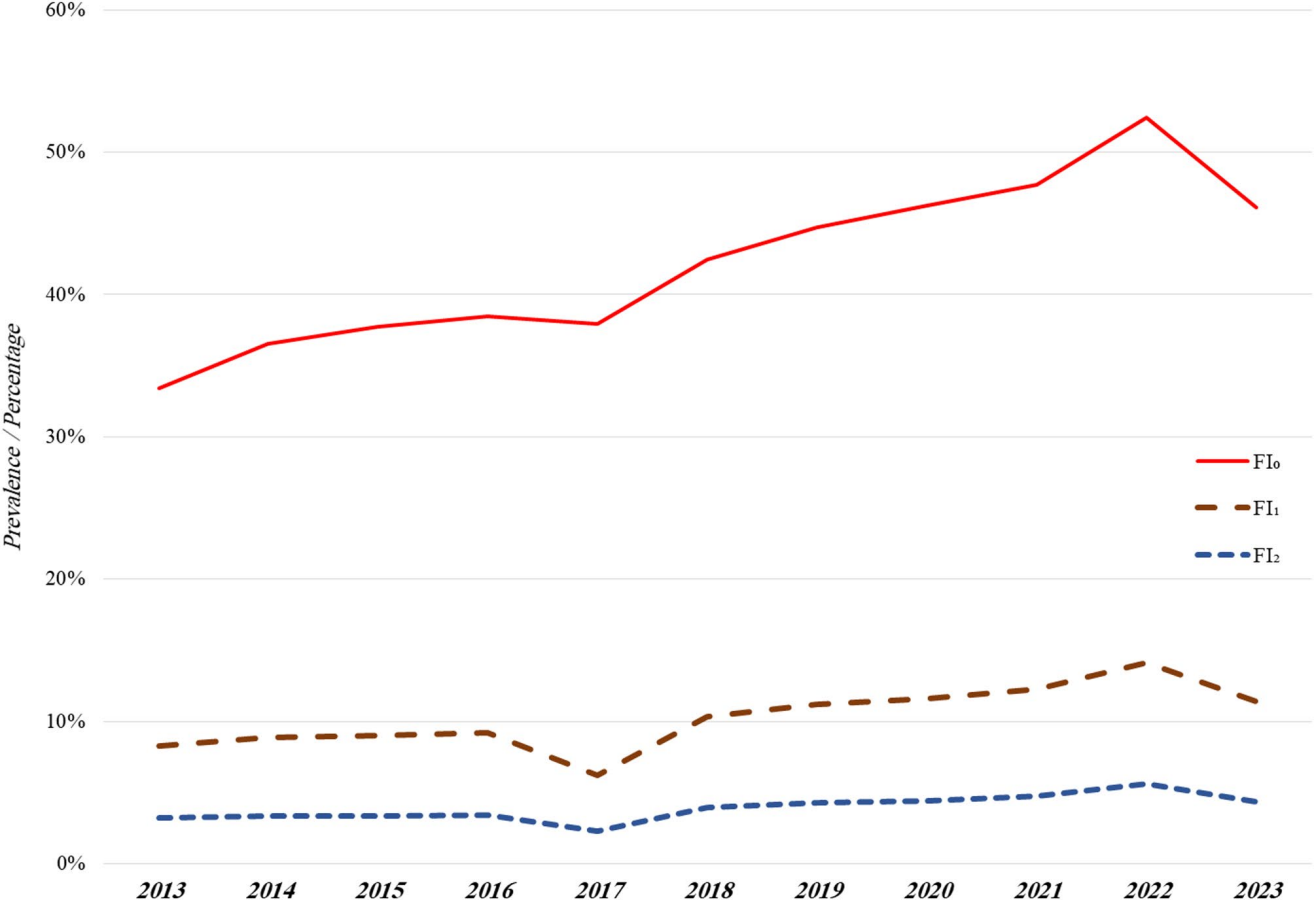
FGT Index (Incidence, Gap, Severity) changes in Iran, 2013–2023: Graph showing the Temporal Trend of the prevalence, insecurity gap, and severity of food insecurity in decade 2013–2023 in Iran.

The FI₀ for 2013–2023 shows a clear upward trend in food insecurity in Iran, rising from 33.4% to 46.1%, a 38% increase in households consuming below the recommended daily caloric intake. Over this decade, more than half of the population experienced food insecurity. Despite the upward trend, low variability (SD ± 8%) indicates persistently high levels. By settlement type, prevalence increased from 37% to 47% in urban areas and from 30% to 45% in rural areas, reflecting a widespread and relatively uniform escalation in household vulnerability (Fig. 2, FI₀).

### Spatial and temporal variation

To ensure 100% population coverage and reproducibility, counties were classified into three mutually exclusive groups:

- **Severe** (µ + SD; Red scheme).
- **Moderate** (µ ± ½ SD; Pale Yellow).
- **Limited** (µ − SD; Blue scheme).

A comparative analysis of FI₀ across Iran’s 31 provinces during 2013–2023 demonstrates marked spatial heterogeneity. Provincial prevalence ranged from 12% to 83%, with an overall mean close to 50%, indicating that approximately half of the population was food insecure during the study period. Temporally, FI₀ generally escalated, surpassing 70% in several regions, highlighting critical worsening conditions.

In total, in order to facilitate the understanding of the spatial pattern of food insecurity, counties can be classified into three groups: Group 1 includes counties facing severe food insecurity where over µ + SD of households in each year receive less than 2100 calories per day (areas with Red scheme); Group 2 comprises counties facing moderate food insecurity where µ ± ½ SD of households in years 2013 to 2023 receive less than the daily standard (areas with Yellow); and Group 3 consists of counties with low food insecurity where less than µ − SD of sampled households receive below the daily standard (areas with blue scheme).

Counties in Tehran, Qazvin, Kerman, Gilan, and Hormozgan consistently reported very high food insecurity rates (> 70%), identifying them as particularly vulnerable. Conversely, Kermanshah and South Khorasan maintained relatively lower prevalence levels (< 35%), reflecting comparatively better food security. Certain provinces, such as West Azerbaijan, exhibited medium-to-high fluctuations, suggesting localized shocks or instability in food access. Overall, the spatial distribution of FI₀ is highly uneven, with persistent clusters of vulnerability and limited resilience in most regions. In 2023, the lowest prevalence was recorded in Semnan (25%), while the highest occurred in West Azerbaijan (65.8%), followed by Hormozgan and Gilan (≈ 60%). Across the decade, Tehran Province exhibited the highest mean food insecurity (57%), whereas Kermanshah Province reported the lowest (19%).

In 2013, 65 of 431 counties were categorized as severely food insecure, including Tehran, Qom, and parts of Alborz, Kerman, Hormozgan, Mazandaran, and Qazvin (> 60%). An additional 50 counties recorded prevalence between 48% and 59%. By 2023, the number of severely food-insecure counties had risen to 73, and more than 200 counties experienced severe insecurity at least once during the decade.

**Fig. 3:**
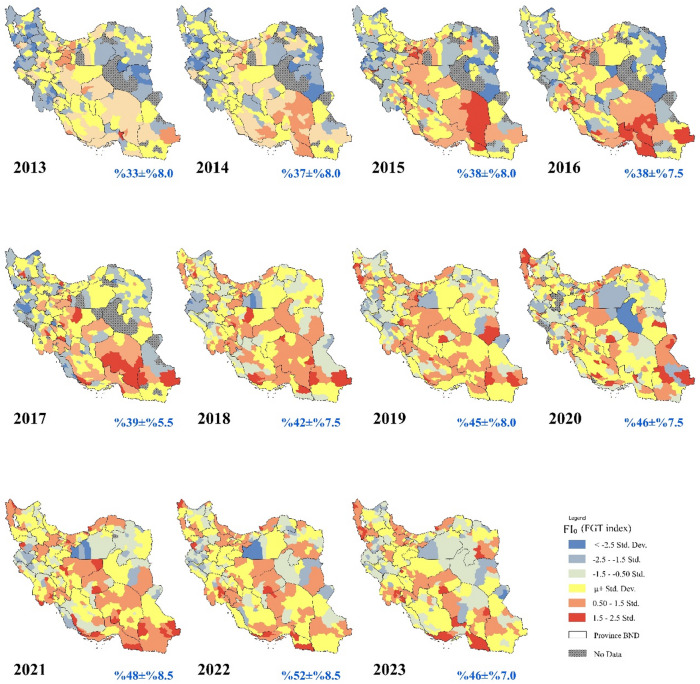
Spatial distribution of FI₀ at the counties, 2013–2023: FI₀ in county level in each year with annual 𝜇 and SD to show the counties with FI₀ greater/equal & smaller than national FI₀. This figure was created using the GeoDa (1.22) and QGIS (version 3.44).

The temporal pattern is noteworthy: from 2013 to 2017, the number of severely affected counties declined to fewer than 50, followed by a sharp increase to over 200 counties by 2023 (Fig. 3).

### Provincial and county-level patterns

**Fig. 4:**
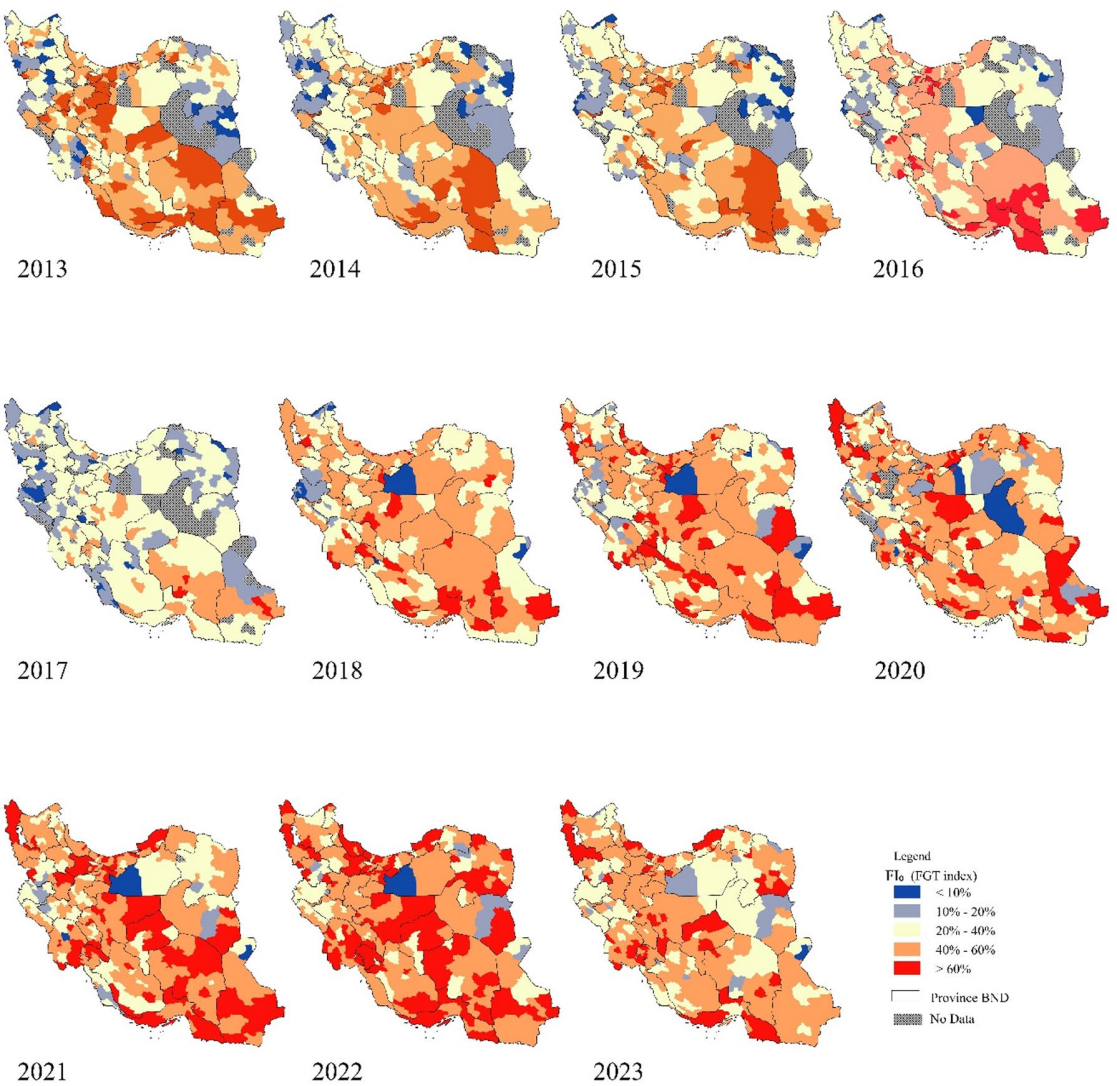
Changes in FI₀ relative to 2013, 2014–2023: Changes in the index FI₀ from the years 2014 to 2023 compared to the beginning of the period, indicating an increase in the number of households facing food insecurity. This figure was created using the GeoDa (1.22) and QGIS (version 3.44).

County-level FI₀ maps from 2013 to 2023 reveal a marked rise in food insecurity. In 2013–2015, most counties showed moderate prevalence, with few exceeding 60% (red zones). From 2018, severe food insecurity expanded sharply in southern provinces (Hormozgan, southern Kerman, Sistan and Baluchestan) and parts of West Azerbaijan, while northern and northeastern counties (Semnan, northern Tehran, parts of Khorasan) remained below 40%, representing cold spots. Central provinces exhibited fluctuating moderate-to-high prevalence, reflecting localized shocks. By 2023, severe food insecurity (> 60%) affected a substantial portion of counties, indicating persistent spatial clustering of vulnerability and limited resilience across much of the country (Fig. 4).

### Food insecurity gap

The analysis of FI₁ across provinces and counties over the study period reveals significant temporal and regional variations, as well as notable differences between the average calorie intake of surveyed households and the standard threshold of 2,100 kilocalories. On average, the FI₁ index was 10%, indicating that food-insecure households consumed approximately one-quarter less than the minimum caloric requirement. The spatial distribution of FI₁ further indicates moderate to high severity of food insecurity in multiple counties, with persistent clusters of high-deprivation areas (see Fig. 2. FI₁).

The FI₁ index ranged from a minimum of 2.4% in Southern Khorasan province in 2017 to a maximum of 19% in Hormozgan province in 2022, highlighting pronounced inequalities in household food security across regions and years. Temporal trends can be summarized as follows:

- **Early years (2013–2016)**: A majority of counties reported relatively low FI₁ values, reflecting comparatively lower severity of food insecurity (National Mean FI₁ < 8.5%).
- **Mid-period (2017–2019)**: Several regions, particularly Tehran and Kohgiluyeh and Boyer-Ahmad provinces (FI₁=16% in 2019) and Lorestan province (FI₁ = 14.5%), experienced sharp increases in food insecurity severity (National Mean FI₁ < 9.3%).
- **Recent years (2020–2023)**: Most regions witnessed further increases in FI₁ values, with peaks in Hormozgan (19%), Kohgiluyeh and Boyer-Ahmad (18.2%), and Gilan (17.9%), highlighting the intensification of deprivation among food-insecure households (National Mean FI₁ < 12.4%).

Spatial disparities are also evident:

- Counties in Hormozgan, Mazandaran, Kordestan, Khuzestan and West Azarbaijan consistently exhibited FI₁ values above 20% in recent years, indicating high vulnerability.
- In contrast, provinces such as South Khorasan, Ilam, Bushehr and Kermanshah frequently reported FI₁ values below 4%, suggesting greater resilience and lower severity of food insecurity.

**Fig. 5:**
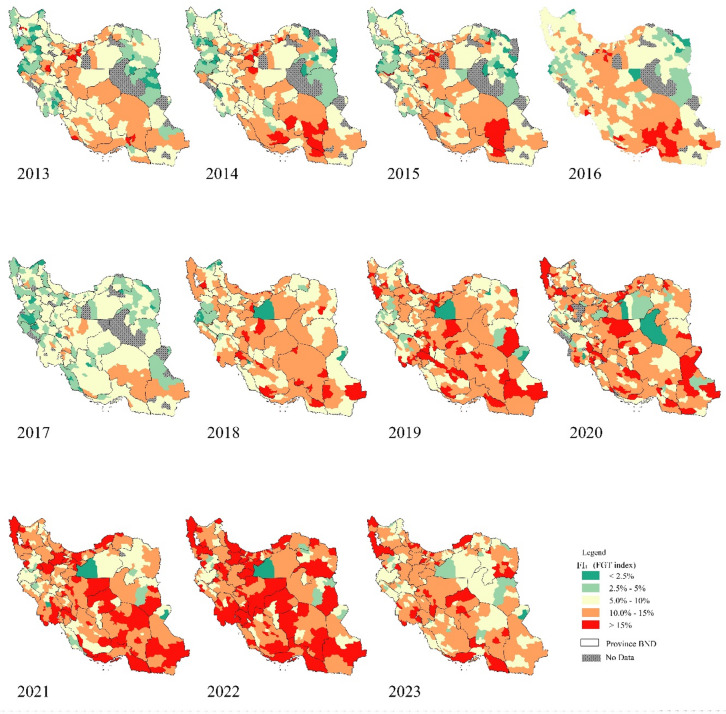
Spatial distribution of FI₁ across counties, 2013–2023: Variation in the FI₁ have gradually increased in decade, indicating that in 2022, the maximum number of counties facing a deficit of more than 15% of the 2100 calories per day was reached. This figure was created using the GeoDa (1.22) and QGIS (version 3.44).

Collectively, these findings underscore the uneven spatial distribution of food insecurity, revealing regions with chronic deprivation alongside areas that remain comparatively less affected. The results indicate a worsening trend in food insecurity severity throughout the study period, coupled with persistent spatial inequality (Fig. 5).

### Food insecurity severity

In Iran, the FI₂ index averaged approximately 3.9% between 2013 and 2023, indicating a moderate level of food insecurity during this decade. However, temporal analysis reveals a worsening trend: severity rose from 3.2% at the beginning of the period to over 5.5% by 2022 and 4.3% in 2023, highlighting an intensification of deprivation in recent years (see Fig. 2. FI₂).

### Spatial patterns

The spatial distribution of FI₂ demonstrates three major patterns:

**1. High-Intensity Provinces**

- **West Azerbaijan and Qom**: In 2023, five counties in West Azerbaijan reported FI₂ values exceeding 7%, with a provincial average above 6%, marking the highest observed severity nationwide. Qom also displayed high FI₂ levels in the early years, which subsequently declined to below 5% by 2022–2023.
- **Hormozgan and Hamadan**: Both provinces recorded sharp increases after 2018, with FI₂ values rising from around 3% in 2017 to over 7% in 2022.
- **Yazd**: This province consistently exhibited high and persistent food insecurity, with FI₂ increasing from 4% in 2013 to over 6% in 2023, reflecting long-term deterioration.

These provinces represent critical hotspots of escalating food insecurity and require targeted support and intervention programs.

**2. Low-Intensity Provinces**

- **South Khorasan**,** Khuzestan**,** and Kermanshah**: These provinces generally maintained FI₂ values below 2%, with only minor fluctuations. • South Khorasan reported the lowest recorded intensity at 0.9% in 2013.
- **Lorestan**: Similarly, FI₂ values remained below 3%, indicating relative resilience.

These provinces demonstrate greater stability and less inequality in food access compared to national trends.

**3. Fluctuating Provinces**

- **Alborz**,** West Azerbaijan**,** Sistan and Baluchestan**,** and Kohgiluyeh and Boyer-Ahmad**: These regions exhibited significant temporal variability. For instance, West Azerbaijan experienced a steady rise in FI₂ from 1.6% in 2013 to 6.7% in 2022, signaling gradual intensification.
- **Qazvin and Kerman**: Both provinces showed sharp interannual fluctuations, including steep declines followed by rebounds, reflecting local shocks and unstable food access at the sub-provincial level.

### Temporal dynamics

- **2013–2016**: Most provinces recorded FI₂ values below the national average (3.4%).
- **From 2017 onwards**: A marked increase occurred, with several provinces exceeding 1.5%, significantly above the national average of 2.2%.
- **2018–2023**: Intensification was most pronounced in West Azerbaijan, Hormozgan, Hamadan, and Yazd, indicating a structural weakening of food security conditions (4.6%).

Overall, FI₂ trends highlight persistent—and in many areas escalating—food insecurity. While provinces such as North Khorasan, Khuzestan, Lorestan, and Kermanshah demonstrate relative resilience, others, notably West Azerbaijan, Qom, Hormozgan, Hamadan, and Yazd, emerge as clusters of critical vulnerability. Addressing these disparities requires equity-oriented interventions to ensure fair access to basic food needs and mitigate both the prevalence and severity of deprivation.

### FI₂ trends

The FI₂ increased from 3.2% in 2013 to 4.4% in 2023, peaking at 5.6% in 2022. Urban-rural disparities are notable: while rural areas remained relatively stable, urban areas experienced more than a twofold increase, particularly between 2022 and 2023, suggesting compounded calorie deficits among urban households (see Fig. 2, FI₂).

**Fig. 6:**
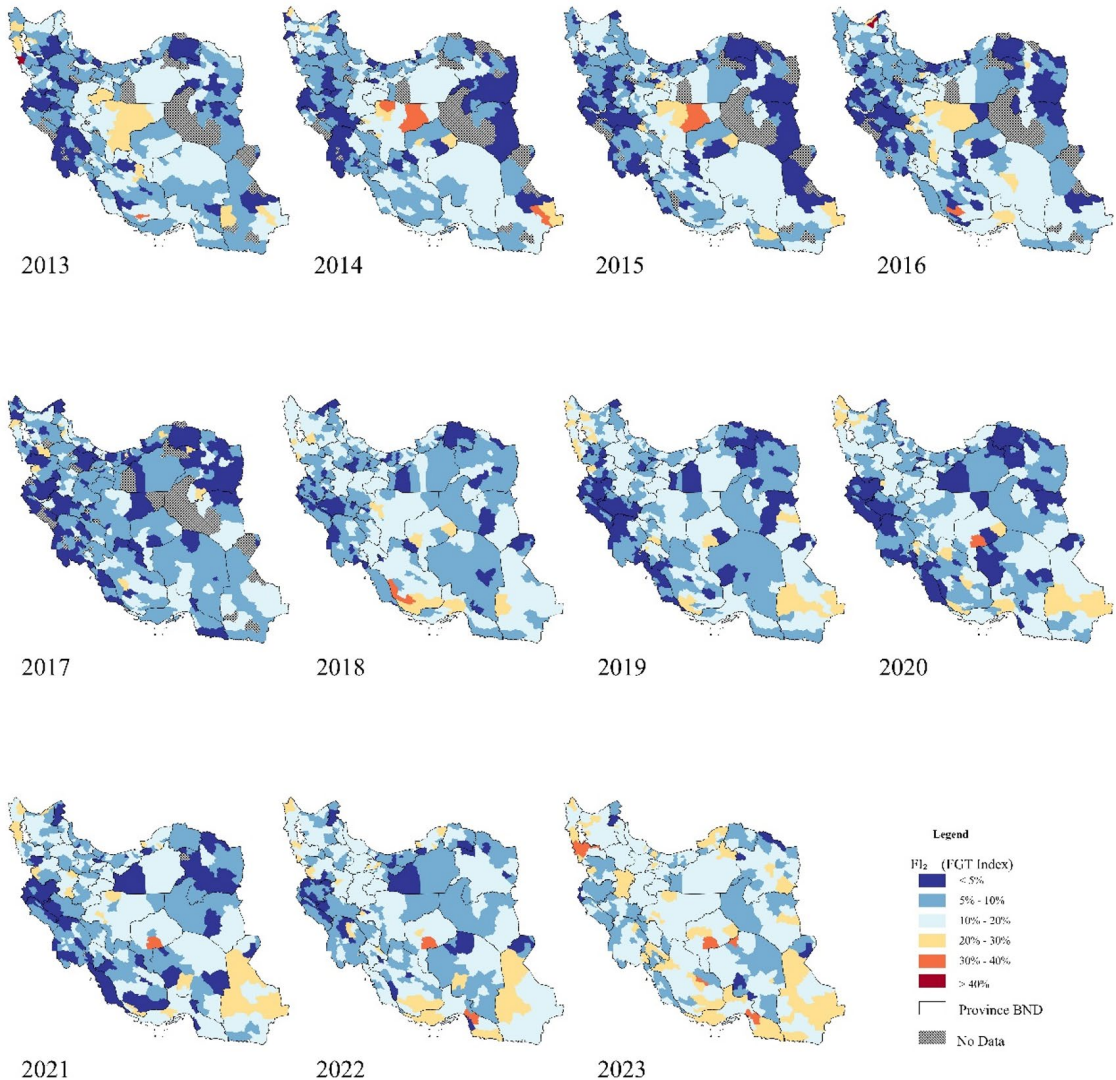
Trends of FI₂ in 2013–2023: The FI₂ was less than 10% in the early years and gradually decreased with a downward trend until 2017. However, it then increased with an upward trend to over 20% in most counties by 2023. This figure was created using the GeoDa (1.22) and QGIS (version 3.44).

Spatial inequality also intensified. In 2013, FI₂ ranged from a maximum of 5.8% (Tehran, Qom) to a minimum of 1% (South Khorasan). By 2023, the range shifted to 6.2% (West Azerbaijan) versus 2.4% (Semnan). Over the ten-year period, the mean FI₂ was highest in Tehran (4.5%) and lowest in Kermanshah (1.8%) (Figure. 6).

### Spatial patterns of food insecurity

The spatial distribution of FGT indicators, analyzed using Moran’s Index, reveals persistent and significant spatial clustering over the ten-year study period. Although the overall spatial concentration increases from 0.376 in 2013 to 0.392 in 2023, notable clusters for both indicators remain evident. Moderate positive spatial autocorrelation is observed in most years, with Moran’s I values ranging between 0.32 and 0.45. This indicates that neighboring units tend to exhibit similar values, suggesting a degree of clustering. The highest clustering is recorded in 2018 (0.447) and 2019 (0.427), indicating stronger spatial dependence. A notable decline occurs in 2020 (0.150), reflecting a temporary weakening of spatial clustering and suggesting more spatial randomness in that year. Post-2020, Moran’s I values rebound to the 0.34–0.40 range, showing the re-emergence of moderate clustering patterns.

Between 2013 and 2018, Moran’s Index consistently exhibited high values, indicating stable and concentrated clusters of food insecurity. In 2020 onward, however, a downward trend emerged, reaching a minimum of 0.15. This decline reflects a gradual weakening of spatial clustering, attributable to three interrelated factors:

1. An increase in the number of individuals experiencing food insecurity,
2. Growing uniformity in the standard calorie gap, and.
3. The transformation of concentrated clusters into more scattered patterns (Table 2).

**Table 2:** Moran’s Index for FI₀, 2013–2023.

Moran’s I	2013	2014	2015	2016	2017	2018	2019	2020	2021	2022	2023
	0.376	0.389	0.323	0.395	0.324	0.447	0.427	0.150	0.397	0.344	0.392
Z Score	11.91	12.96	10.78	12.16	10.81	14.86	14.20	4.80	13.18	11.45	13.06
P value	0.00	0.00	0.00	0.00	0.00	0.00	0.00	0.00	0.00	0.00	0.00

**Fig. 7:**
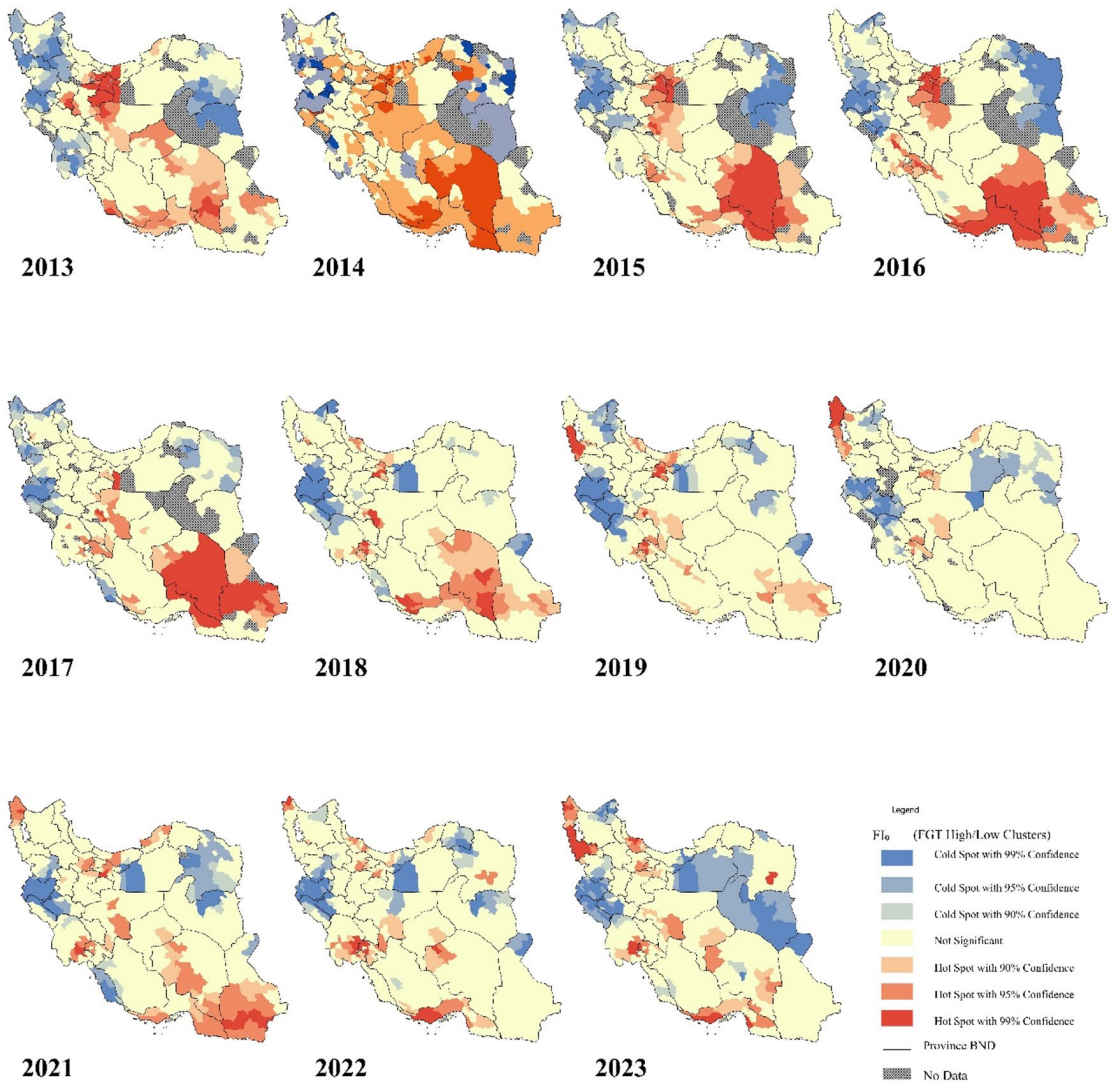
Local Spatial Autocorrelation of FI₀, 2013–2023: The spatial pattern of clusters of FI₀, which has shifted from a concentrated state with limited clusters to a relatively uniform state across the counties throughout the 10 years, signifies an increase in food insecurity in most counties of the country towards the end of the study period. This figure was created using the GeoDa (1.22) and QGIS (version 3.44).

Policy shifts played a decisive role in shaping these spatial patterns. In 2016, the elimination of certain food and goods subsidies contributed to a sharp rise in FI₀, which reached 51%. A subsequent policy shock in 2022—the removal of subsidies for four key commodities (flour, poultry and eggs, sugar, and oil)—further escalated food insecurity, raising FI₀ above 62%. The decline in Moran’s Index during these periods reflects the geographic expansion of food insecurity, resulting in more dispersed clusters rather than localized concentrations (Figure 7).

Results from the local spatial autocorrelation analysis FI₀ provide additional insights. At the beginning of the study period (2013), limited but statistically significant clusters of counties fell below the standard daily caloric intake of 2,100 kilocalories. These clusters were primarily located in central provinces (Tehran, Qom, Markazi, and Isfahan) and sporadically in Kerman, Sistan and Baluchestan, Fars, Hormozgan, and Bushehr. By 2017, the extent of food-insecure areas expanded considerably, encompassing many more counties within these regions.

During 2013–2016, as food-insecure counties (depicted in red patches) increased, relatively food-secure areas in the northwest, west, and east diminished. In 2018 and 2022, government policies that removed subsidies while offering compensatory cash transfers temporarily reduced the concentration of clusters. Nevertheless, these measures also intensified widespread food insecurity due to rising household food basket costs.

From 2018 to 2023, the remaining clusters were concentrated in counties experiencing severe food insecurity, where the food insecurity gap exceeded 30% below the standard caloric threshold.

## Discussion

This study presents a decade-long, county-level spatiotemporal assessment of food insecurity in Iran, revealing a trajectory characterized by rising prevalence, increasing severity and widening spatial inequalities. By integrating micro-level household data with spatial analytical approaches, the findings provide a more granular perspective than conventional macro-level assessments and expose important discrepancies between national aggregates and lived household realities.

A central finding is the systematic divergence between micro-level Household Income and Expenditure Survey (HIES) data and FAO macro-level estimates. While global monitoring frameworks often suggest relative stability or modest improvements in food security indicators, the results here indicate a marked deterioration. This discrepancy reflects a key limitation of macro-level metrics, which are insufficiently sensitive to localized inflationary pressures and rising non-food expenditures—particularly housing and energy costs—that directly constrain household food access^2^^,37^. These findings reinforce the understanding of food insecurity as a multidimensional phenomenon shaped not only by food availability but also by economic access and purchasing power^38^.

Placed within a global context, the observed trends are consistent with patterns documented across low- and middle-income countries, where economic instability, inflation and structural inequalities have intensified food insecurity in recent years6–8. Although recent global assessments report slight improvements in aggregate indicators, longer-term trends point to increasing vulnerability, with substantial growth in the number of food-insecure individuals worldwide^3^. Evidence from sub-Saharan Africa and South Asia consistently identifies rising living costs, income volatility and policy shocks as key drivers^39^. Iran’s trajectory closely mirrors these dynamics, particularly among middle-income countries experiencing macroeconomic pressures combined with policy-induced disruptions, where national averages obscure substantial subnational disparities.

At the national level, prior studies have highlighted the importance of socioeconomic determinants—including household size, employment instability and income inequality—in shaping food insecurity outcomes ^40-42^. However, much of this literature relies on cross-sectional or aggregated analyses, limiting its ability to capture spatial persistence and temporal dynamics. By contrast, the present study employs a longitudinal, county-level framework that reveals persistent hot spots and evolving spatial patterns, thereby addressing a critical empirical gap.

The findings demonstrate a substantial increase in food insecurity prevalence (FI₀), rising from 33.4% in 2013 to 46.1% in 2023. The persistence of elevated FI₀ levels across the study period suggests the presence of a structural baseline vulnerability, whereby a considerable share of households remains chronically exposed to food insecurity even during relatively stable periods. This pattern is consistent with international evidence indicating that food insecurity is often structurally embedded within economic systems characterized by inequality and limited social protection^38^^,39^. The broadly similar trends observed across urban and rural areas further suggest that nationwide macroeconomic forces, rather than purely localized shocks, have been the dominant drivers.

The spatial analysis reveals pronounced and persistent geographical inequalities in food insecurity, with specific counties forming high-risk clusters over time. Statistically significant Moran’s I values confirm strong spatial dependence, indicating that food insecurity is not randomly distributed but instead reflects underlying regional disparities in economic opportunity, infrastructure and access to services^36^. A notable feature is the temporary decline in spatial clustering around 2020, coinciding with major economic disruptions. This pattern likely reflects a diffusion effect, whereby widespread shocks extend food insecurity beyond established hot spots into previously less-affected regions. Similar dynamics have been observed in other contexts, where systemic shocks generate more spatially homogeneous vulnerability patterns^43^. At the same time, the persistence of certain high-risk clusters suggests entrenched structural disadvantages that cannot be explained solely by short-term fluctuations, underscoring the need for place-based policy responses.

Beyond prevalence, the analysis of FI₁ and FI₂ indices indicates a clear intensification of food insecurity. The average FI₁ reflects a substantial caloric deficit among affected households, while rising FI₂ values point to increasing inequality in food access, with the most vulnerable populations experiencing disproportionately severe deprivation. These findings highlight the importance of moving beyond binary measures toward indicators that capture depth and distributional inequality^44^. Importantly, the spatial distribution of severity does not fully coincide with that of prevalence, suggesting that regions with moderate overall prevalence may still contain populations facing acute nutritional deficits. This has direct implications for policy targeting, as reliance on prevalence alone may overlook pockets of extreme deprivation.

One of the most striking findings is the temporal alignment between sharp increases in food insecurity indices and major subsidy reforms in 2016 and 2022. Although the analysis remains descriptive and does not establish causality, the consistency and magnitude of these associations strongly suggest that policy-induced price shocks played a critical role. The 2022 reform, in particular, corresponds with a marked surge in both the prevalence and spatial extent of food insecurity. While compensatory cash transfers were implemented, the results indicate that these measures were insufficient to offset the rapid erosion of purchasing power caused by rising food prices and broader inflationary pressures. This outcome is consistent with international evidence showing that subsidy removal policies, when inadequately designed or implemented, can have regressive effects and disproportionately impact low-income households^45,^^46^.

These findings have several implications. First, they highlight the limitations of relying on aggregate indicators and underscore the importance of incorporating high-resolution, spatially explicit data into food security monitoring systems. Second, they emphasize the central role of purchasing power and cost-of-living dynamics in shaping food access, suggesting that stabilizing real incomes and controlling inflation may be as critical as interventions targeting food supply. Third, they demonstrate the value of integrating household-level data with spatial analysis to identify persistent vulnerabilities and inform targeted, region-specific policies.

Several limitations should be acknowledged. The analysis is primarily descriptive and exploratory, and although strong temporal and spatial associations are identified, causal relationships cannot be definitively established. The reliance on secondary data may introduce measurement constraints, particularly in capturing informal economic activities and intra-household food allocation. In addition, caloric-based indicators do not fully reflect diet quality and nutritional diversity, which are essential dimensions of food security. Future research could build on these findings by applying causal inference approaches, incorporating higher-frequency data and integrating qualitative methods to better understand household coping strategies and the mechanisms linking economic shocks, policy interventions and food security outcomes.

## Conclusion

This study provides robust evidence that food insecurity in Iran has evolved into a structural, spatially uneven and intensifying challenge, driven by the interaction of economic pressures, policy shifts and regional inequalities. Over the past decade, food insecurity has followed a clear upward trajectory with no indication of reversal, accompanied by persistent geographical clustering and a marked deepening in severity. The concentration of high-risk areas alongside the expansion of vulnerability into new regions highlights the dual nature of the problem: entrenched local deprivation coexisting with increasingly widespread exposure to economic shocks.

The findings demonstrate that food insecurity is highly responsive to macroeconomic conditions and policy interventions, particularly subsidy reforms, which appear to have amplified household vulnerability by eroding purchasing power. The more rapid intensification observed in urban areas further underscores the role of rising living costs and structural economic constraints in shaping access to food. Together, these patterns reinforce the need to conceptualize food insecurity not as a temporary or isolated phenomenon, but as a systemic outcome of interconnected economic and spatial processes.

From a policy perspective, the persistence of spatial inequalities and the emergence of new hot spots point to the limitations of uniform, nationwide approaches. Effective responses will require targeted, place-based interventions that prioritize high-risk regions, alongside strengthened social protection systems capable of buffering households against economic shocks. In particular, safeguarding purchasing power, improving the design and timing of policy reforms, and enhancing the resilience of local livelihoods will be critical to mitigating further deterioration. The integration of spatially explicit monitoring systems into national food security frameworks could also support more timely and adaptive decision-making.

Although focused on Iran, these findings have broader relevance for countries experiencing economic volatility, subsidy reforms or widening inequality. Three generalizable patterns emerge: the amplification of food insecurity following policy-induced economic shocks, the persistence of spatial hot spots, and the intensification of vulnerability in urban contexts. These insights highlight the importance of combining high-resolution spatial analysis with household-level data to better anticipate emerging risks and guide resource allocation in complex and rapidly changing environments.

Several limitations should be considered. The reliance on survey-based caloric intake data may introduce reporting bias, and the county-level scale does not capture intra-county or intra-household heterogeneity. In addition, although the temporal alignment between policy changes and food insecurity trends is suggestive, causal relationships cannot be definitively established. The annual resolution of the dataset also limits the detection of short-term fluctuations, and key factors such as informal food systems, migration dynamics and climate-related shocks were not explicitly modelled.

Future research should aim to integrate higher-frequency and higher-resolution data, including detailed socioeconomic and environmental indicators, to better capture the multidimensional nature of food insecurity. Applying causal inference approaches and modelling interactions between policy interventions and local food systems would further strengthen understanding of underlying mechanisms and resilience pathways.

Without sustained and well-targeted policy responses, the spatial disparities and nutritional deficits identified in this study are likely to intensify, with important implications for public health, social stability and long-term development.

## Data Availability

The HIES data are available via at https:// [https://amar.org.ir/](https:/amar.org.ir) (ref. 30). The map of province and counties are available on request from Interior ministry of Iran.

## Footnotes

1 Classification of Individual Consumption According to the Purpose.
